# Preventive bundles to reduce catheter-associated bloodstream infections in neonatal intensive care

**DOI:** 10.3205/dgkh000316

**Published:** 2018-11-16

**Authors:** Sarah Schmid, Christine Geffers, Gudrun Wagenpfeil, Arne Simon

**Affiliations:** 1 University Hospital of the Saarland, Children’s Hospital, Pediatric Oncology and Hematology, Homburg, Germany; 2 German National Reference Center for Surveillance of Nosocomial Infections, Institute for Hygiene and Environmental Medicine, Charité-Universitätsmedizin Berlin, Germany; 3 Institute for Medical Biometrics, Epidemiology and Medical Computer Sciences, University Hospital of the Saarland, Homburg, Germany

**Keywords:** preterm infants, neonatal intensive care, central-line associated bloodstream infection, preventive bundle

## Abstract

This systematic survey includes a total of 27 studies published between 2002 and 2016 on the benefit of preventive bundles for the prevention of central-line associated bloodstream infections (CLABSI) in neonatal intensive care. These studies are mainly cohort studies or studies analyzing an interrupted time series before and after intervention. The studies showed heterogeneity in terms of endpoint definitions (CLABSI), details of the implemented measures, and evidence of a publication bias favoring the use of of preventive bundles. The cumulative analysis showed a statistically and clinically significant benefit of preventive bundles to avoid CLABSI in neonatal intensive care.

## Zusammenfassung

In einer systematischen Übersicht zu neonatologischen Intensivpatienten mit zentralem Venenkatheter wurden die Ergebisse von insgesamt 27 Studien ausgewertet, die den Nutzen von Präventionsbündeln zur Vermeidung von katheterassoziierten Blutstrominfektionen (CLABSI) untersucht haben. Die eingeschlossenen Studien wurden zwischen 2002 und 2016 publiziert. Die meisten Studien waren Kohortenstudien oder unterbrochene Zeitserien vor und nach Einführung von Präventionsbündeln. Entsprechend heterogen sind die Definitionen der Endpunkte und die unterschiedlichen präventiven Maßnahmen; zudem gab es Hinweise auf einen Publikationsbias zugunsten von Präventionsbündeln. Trotz dieser methodischen Limitationen zeigen die analysierten Studien einen signifikanten und klinisch sehr relevanten Nutzen von Präventionsbündeln in der neonatologischen Intensivpflege.

## Background

Preterm infants and neonates in intensive care bear a high risk for nosocomial infections (NI) [1]. Level 1 and level 2 highest-care NICUs are among those risk areas where selected NI are monitored prospectively, in addition to monitoring of invasive pathogens and their antibiotic resistance profiles [2]. The findings serve to improve patient safety and quality of treatment by preventing NIs, preventing infection by multiresistent pathogens, and optimizing the use of antibiotics [3]. Local findings of the German NEO-KISS monitoring can be checked against anonymized reference data [4], [5], [6], [7], [8]. For example, there is a positive effect of prospective monitoring on the reduction of central-line associated bloodstream infections (CLABSI) [9].

The use of central (CVC) and peripheral venous catheters (PVC) has been identified as an indepentent risk factor for late-onset sepsis (LOS) in NEO-KISS participants [7]. NICUs mostly use umbilical vein catheters (UVC) and peripherally inserted central venous catheters (PICC) as central lines. Analysis and evaluation of NEO-KISS data yield important information on quantity, etiology, and pathogen range of CLABSI [10], [11]. Between January 2012 and December 2016, the median CLABSI rate (incidents per 1,000 CVC utilization days) in preterm infants was 8.62 at a birth weight (BW) of 499 g, 5.29 at BW 500 to 999 g 5.29, and 2.35 at BW 1,000 to 1,499 g. Thus, NICUs show a significantly higher CLABSI rate than pediatric ICUs [12]. Fortunately, the CLABSI rate of preterm infants with a birth weight below 1,500 g (very low birth weight, VLBW) has decreased continuously for years now. Between 2007 and 2011, PVC-associated sepsis rates were nearly constant between 6.7 and 7.5 per 1,000 PVC utilization days [4], but most recently, between January 2012 and December 2016, the median rate per 1,000 PVC utilization days was 3.44 in ELBW preterm infants (birth weight between 500 and 999 g) and 2.18 in VLBW preterm infants (birth weight 1,000 to 1,499 g).

In 2007, the German Commission for Hospital Hygiene and Infection Prevention (KRINKO) published a recommendation for the prevention of nosocomial infections in NICU patients [13], comprising explicit recommendations for the prevention of infections associated with central lines. An update has been published recently [14] in order to support specialist NICU teams in reviewing and sustainably implementing their local standard of CLABSI prevention [15]. 

To merge single measures e.g. from national guidelines into an individual bundle for each hospital may lead to a significant improvement in treatment quality in the long term [16], [17], [18], [19], [20], [21], [22], [23], [24], [25], [26], [27], [28], [29], [30], [31], [32], [33], [34], [35], [36], [37], [38]. 

The aim of this systematic investigation was to evaluate the available studies on the use of preventive bundles for the prevention of CLABSI in NICUs. This should lead to a better basic understanding of the benefits of preventive bundles in this special context and point to the characteristics of this patient population.

## Methods

We searched papers in PubMed (last search Oct. 1, 2016; key words “central venous line, neonatal intensive care, prevention, preventive bundle, central line-associated bloodstream infection”) and included secondary citations found in these articles and surveys to find clinical studies which were published according to peer-review procedures in Medline-listed scientific journals between 2002 and 2016. Eligible studies contained precise information on the most important aspects of infection prevention when inserting or handling central venous lines in neonatal ICU patients. Moreover, they had to present the method of diagnosing CLABSI, endpoint definitions and the effect of the preventive strategy, e.g., on the CLABSI rate in %, CLABSI incidences per 1,000 hospitalization days, or the CLABSI incidence rate per 1,000 treatment days. As randomized controlled studies have been performed very rarely in this patient population, we also included studies which used other infectiological-epidemiological methods to compare patient populations with similar basic pre- and postinterventional characteristics (implementation of preventive bundle) [39]. 

The most important information was entered into a structured table of findings. Where applicable, the presented survey pointed out methodological limitations of the studies, keeping in mind the basic limitations of non-prospectively randomized controlled studies.

Eight studies disclosed the pre- and postinterventional CLABSI incidence rate (incidents per 100 patients), 7 studies contained evidence for an incident rate (incidents per 1,000 treatment days), and in both groups, the original publication provided information on the number of patients, number of incidents, or number of treatment days for central lines. We merged the data of this specific selection from the the total number of studies we found into an outlined meta-analysis. We used StatsDirect version 3.0.183 (Nov. 1, 2016) for meta-analysis and for calculating the combined relative risk, with a corresponding 95% confidence interval. Applying the so-called fixed effects model in combination with Cochran’s Q test, the null hypothesis “there is heterogeneity between the studies” was permissible. Forest plots were used for data presentation. Additionally, the findings were analyzed by means of a funnel plot and the corresponding Egger’s Test for Symmetry to show a possible publication bias.

## Results

### Number and methodology of the enclosed studies 

A total of 27 studies were included in this analysis; see survey in Table 1 (Tab. 1) and Table 2 (Tab. 2). The design of the included studies was heterogeneous. There were monocenter retrospective surveillance studies [27], [28], [30], [33], [40], [41], [42], an experimental study [26] and prospective cohort studies [34], [35], [38], [43], [44], [45], [46], [47], [48]. Moreover, we analyzed 10 multicenter studies performed by cooperative surveillance networks [24], [25], [29], [36], [37], [49], [50], [51], [52], [53]. The multicenter studies comprised findings from 6 [53] to 100 NICUs [24] per study. To the authors’ knowledge, no prospectively randomized controlled studies were published on the use of preventive bundles in neonatal ICU patients up to September 2017.

### Definition of incidents 

Most studies [24], [25], [27], [29], [30], [33], [35], [36], [38], [42], [43], [45], [46], [50], [52] use the criteria of the Centers for Disease Control and Prevention (CDC) [54] to define CLABSI. Attention should be paid to the fact that in 2008, there was a change in the CDC’s definition of CLABSI caused by CoNS or other potential blood culture contaminants. From 2008 onwards, two independent blood cultures were demanded for verifying a BSI in every case [54]. When the respective study was not completed in 2008 or when investigation of a required control group took place before 2008, the problem arose of two different definitions during the time of the same study [26], [27], [29], [35], [42], [52]. In 3 studies, the data were corrected retrospectively using the new definititon [26], [35], [52]. One other study kept the previous definition [42]. Two studies [34], [37] used the definitions of the German NEO KISS Module [4]. Two studies [47], [48] did not describe the definition of incidents in detail. 

The primarily documented incidents of some studies were reviewed by independent infectiology/hygiene specialists [30], [33]. Golombek et al. [28] registered blood-culture-negative CLABSI in the case of a clinical worsening with suspected infection and subsequently 7 days of antibiotic treatment, beginning 24 hrs after PICC insertion or within 24 hrs after PICC removal. Finally, there are studies in which the endpoint definitions do not exactly match the ones of CDC or NEO-KISS [28], [40], [41], [44], [49], [51], [53].

### Blood culture diagnostics 

The only detailed description of the blood culture sampling procedure is reported in the paper by Kilbride et al. [53]; in the case of a suspected infection, two peripheral venous blood cultures with a minimum volume of 1 ml were drawn. Most studies contain information on the required number of blood cultures [24], [25], [26], [27], [28], [29], [30], [33], [34], [35], [36], [37], [38], [40], [41], [42], [43], [44], [45], [46], [49], [50], [51], [52], [53], but do not comment on the minimum volume of blood per blood culture bottle.

### Definition of prevention goals 

Seven studies contained clear goals as to what should be accomplished for the safety of patients in a defined period of time by implementing the preventive bundle [25], [36], [47], [48], [50], [51], [53]. The aim of Cooley et al.’s initiative [47] was to reduce catheter-associated infection rates by a minimum of 50% in 12 months, which they achieved. The goal of reducing the CLABSI rate by 75% was not fully met by Fisher et al. [50], who attained 71%. Wilder et al. [48] obtained up to 92% real reduction of CLABSI rate from 2011 to 2014 versus a target rate of at least 50%, whereas Wirtschafter et al. [25] aimed for and reached a 25% reduction of the CLABSI rate. The initial incidence rate of CLABSI at the start of the initiative is a crucial factor for formulating a clear goal. At the start, catheter-associated incidence rates in these studies [25], [36], [47], [48], [50] ranged from 1.16 up to 4.32 CLABSI/1,000 PICC utilization days. 

### Clinical implementation of preventive bundles 

The studies took different approaches to implementing the preventive bundle. In most studies, a higher-ranking, responsible multidisciplinary team [1], [3], [5], [6], [8], [10], [11], [16], [17], [18], [20], [21], [22], [23], [25] of up to 20 members [26] effected the implementation. Ting et al. [42] and Kilbride et al. [53] preferred implementing preventive measures in manageable “plan-do-check-act” cycles [31]. 

McMullan et al. [40] describe a structured training program according to the SCORPIO method [32] for implementing the preventive bundle. SCORPIO requires knowledge transfer and training of practical skills; thus, tutors explain and demonstrate the precise procedure to small groups in a multistep training environment (e.g., CVC insertion, dressing change, IV system change).

### Feedback of surveillance findings and compliance rates to treatment team

Periodic feedback of current CLABSI rates to the treatment team is essential to illustrate the benefits of preventive measures or the initial extent of the problem. Many studies implemented this feedback [25], [26], [27], [29], [30], [33], [40], [44], [48], [49], [50]. McMullan et al. [40] describe a monthly feedback of CLABSI rates to the senior physician, a quarterly feedback to team members during the training progam and a 6-month formal findings report on utilization rates and CLABSI rates. According to Bizzarro et al. [26] and Dumpa et al. [30], reports on interim findings and amount of days without CLABSI were displayed in the staff break room. According to Curry et al. [27], there was positive feedback after 100 CLABSI-free days and staff members were particularly praised (pizza party). Those studies reviewing the staff-member compliance with the preventive bundles kept their staff informed about results by displaying them on notice boards [33], [44] or by distributing a newsletter [48]. Shepherd et al. [29] report that the findings of compliance checks were made accessible in the hospital’s intranet.

### Hand hygiene

Nearly all preventive bundles focussed on hand hygiene [24], [25], [26], [27], [29], [30], [33], [34], [36], [37], [38], [40], [42], [43], [44], [45], [46], [47], [48], [49], [50], [51], [52], [53]. Many preventive bundles [24], [25], [29], [33], [36], [42], [47], [48], [50], [51], [53] contained detailed instructions for hand hygiene. Some studies required the use of new disposable gloves in addition to hand disinfection upon each contact with an infusion system [24]. The compliance with hand hygiene was checked explicitly in 6 studies [33], [42], [43], [47], [49], [53]. Kime et al. [33] monitored hand hygiene compliance continuously. A survey among the medical staff showed that 85% of the staff members were not convinced of the specific benefit of intensified hand hygiene for preventing CLABSI [33]. The implementation of special hand hygiene training modules is mentioned in 10 studies [26], [30], [33], [34], [36], [37], [38], [44], [46], [49]. 

According to some American studies, e.g., Cooley et al. [47], hand washing with an antiseptic soap containing 2% chlorhexidine gluconate (CHG) was performed instead of disinfection of hands with alcohol.

### Critical indication and limitation of indwelling

Some studies discussed a critical indication for inserting PICCs [37], [40]. In order to reduce the duration of indwelling, 8 studies [24], [25], [26], [28], [33], [38], [49], [50] defined criteria for PICC removal as early as possible. The catheter was removed in 6 studies as soon as enteral nutrition was 120 ml/kg/d [24], [25], [33], [38], [49], [50], and Bowen et al. [49] defined an enteral nutrition of 120–140 ml/kg for catheter removal.

### Skin antisepsis

The issue is still unresolved as to which is the most suitable kind of skin antisepsis when inserting a central line in very immature preterm infants, above all in preterms with <1000 g birth weight during the first two weeks of life [1]. Ten out of 27 studies [28], [30], [33], [34], [38], [41], [45], [46], [49], [53] do not give precise information on choice of skin antiseptic. However, the preventive bundles of most studies explicitly specify skin antisepsis before insertion of CVC [25], [27], [29], [36], [40], [42], [44], [47], [50], [52] and recommend certain antiseptics [25], [29], [36], [47], [50], [51], [52]. Six studies report skin antisepsis with chlorhexidine (CHG) before inserting PICCs [25], [27], [36], [40], [47], [50]. Exposure time was said to be 30 sec to 3 minutes, with a longer exposure time when inserting a central line into the femoral vein [29], [36]. CHG concentration in these studies was between 0.015% and 3.15% [25], [29], [36], [40], [42], [44], [47], [48], [52], and the isopropanol concentration was between 4% for combined preparations and 70% [26], [29], [36], [42], [44], [47], [52]. In 6 studies [29], [36], [42], [44], [47], [52], skin antisepsis was effected by CHG 2%/ isopropanol 70%. Five studies used povidone-iodine , [25], [29], [36], [47], [50]. Fisher et al. [50] and Piazza et al. [36] allowed skin antisepsis with isopropanol without CHG. 

At dressing changes, catheter insertion points were disinfected with CHG in 6 studies [24], [27], [35], [43], [48], [51]. Two studies [42], [44] used CHG/isopropanol, 4 studies povidone-iodine [24], [26], [48], [51], and 1 study used isopropanol 70% instead of povidone-iodine [26]. Some studies had restrictions on antiseptic use, depending on birth weight, gestational and chronological age [27], [29], [36], [43], [44], [47]. According to Piazza et al. [36] and Shepherd et al. [29], 70% isopropanol or povidone-iodine was used in premature infants with a chronological age of less than 2 months, while CHG 2%/isopropanol 70% were used when the chronological age was ≥2 months. Cooley et al. [47] state CHG 2%/isopropanol 70% as antiseptic for neonates of ≥28 weeks (GA) and chronological age of ≥10 days, and povidone-iodine for younger neonates. Curry et al. [27] allowed CHG 2%/isopropanol 70% when birth weight was higher than 1,000 g and chronological (postpartal) age was at least 2 weeks. Chandonnet et al. [43] and Neill et al. [44] set the limit for using CHG 2%/isopropanol 70% at a minimum of 28 weeks of pregnancy. Ting et al. [42] stipulated swabbing the antiseptic with sterile saline solution at the end of exposure time in premature infants of BW <1,000 g.

### Maximum barrier precautions when inserting central lines 

The preventive bundles of most studies [25], [27], [29], [34], [35], [36], [37], [38], [40], [42], [43], [45], [47], [49], [50], [51], [52] require protective clothing (sterile gloves, surgical face mask, sterile coat, headgear) and extensive surgical draping of patient. Additionally, Piazza et al. [36], Fisher et al. [50]. Kaplan et al. [51] and Wirtschafter et al. [25] recommend surgical face masks for staff assisting within a 1.5 m range. Headgear is not mentioned in all studies [42] and 10 studies do not give detailled information on preventive measures [24], [26], [28], [30], [33], [41], [44], [46], [48], [53].

### Empowerment of staff 

The assisting staff in 5 studies were entitled to stop catheter insertion when there was evidence of a failure to comply with preventive standards, which could make the insertion procedure not aseptic [24], [25], [42], [50], [51]. This medical staff followed a checklist for the decision to intervene [33], [42].

### Reviewing compliance, checklists, daily goals

The benefit of preventive bundles can only be assessed realistically by checking the compliance with its preventive measures. 20 studies (74%) performed a compliance check [24], [25], [26], [29], [30], [33], [35], [36], [40], [42], [43], [44], [45], [46], [47], [48], [50], [51], [52], [53]. However, methods of monitoring and feedback varied widely. Specific inspection of hand hygiene was most frequent [25], [29], [33], [36], [40], [42], [43], [44], [47], [49], [53]. As part of the intervention, most studies [25], [29], [30], [33], [34], [35], [36], [37], [40], [42], [44], [45], [46], [50], [52] have checklists for catheter insertion and maintenance. Shepherd et al. [29] evaluated the compliance with preventive measures for inserting and maintaining catheters through independent monitoring according to checklists. After one year, the compliance with the preventive protocol for insertion and maintenance of catheters was constantly above 90%. Kaplan et al. [51] described a monthly check of compliance with each measure of the preventive bundle; compliance was over 90% in 24 NICUs, but there were also centers with lower compliance. 15 studies [24], [25], [26], [29], [33], [35], [36], [37], [38], [40], [43], [49], [50], [51], [52] used “daily goal sheets”. These are standardized forms to check and discuss critical control points during daily rounds, above all the question of whether the CVC must remain in situ or can be removed.

### Dressing changes

Dressing changes are another keystone in preventing CLABSI. Dressing changes can be effected under extended barrier precautions [24], [25], [26], [29], [35], [36] or aseptically [27], [41], [52]. Holzmann-Pazgal et al. [35] describe extended barrier precautions for dressing changes; in addition to hand disinfection, the staff wore headgear, surgical face masks, sterile coats and sterile gloves. This was similar to Piazza et al. [36]. 

In some studies [26], [36], [41], [43], [44], [48], two persons were required for changing dressings. 

In addition to these differences in daily practice, the studies showed no uniform dressing change interval. In 9 studies [26], [28], [33], [40], [44], [46], [47], [49], [52], the semipermeable transparent film dressing of PICC was changed only when the dressing was contaminated, no longer intact or tending to detach. Curry et al. [27] implemented a weekly change of Broviac line dressing, including CHG-releasing sponges and a change of PICC dressings every 2 weeks. Not all protocols reported intervals for dressing changes [24]. Curry et al. [27] used CHG-releasing sponges to cover PICC insertion points in infants of at least 28 weeks gestational age and at least 10 days of chronological age. In individual patients with skin irritation, the CHG-releasing sponges were replaced by a silver-alginate dressing. The latter was used by Neill et al. [44] as well.

### Change of infusion system

The preventive bundles of 13 studies give detailed instructions on changing procedures for infusion systems [25], [30], [33], [35], [36], [41], [42], [44], [47], [48], [51], [52], [53]. Only 2 protocols [41], [48] required 2 persons for a change. Eight studies [25], [30], [36], [41], [42], [44], [47], [53] recommended changing infusion systems at regular intervals. 

For example, Aly et al. [41] changed systems daily when lipid solutions, blood or blood products were administered. Short infusion systems were removed directly after administration. Neill et al. [44] changed the system every 96 hrs when administering cristaloid solutions without lipids. Ting et al. [42] and Kilbride et al. [53] changed infusion systems every 72 hrs. (in case of blood transfusion within 24 hrs). Dumpa et al. [30] and Cooley et al. [47] recommended changing the infusion system every 24 hrs.

### Pre-assembled flushing syringes 

As part of the preventive bundle, pre-assembled flushing syringes with sterile physiological saline solution were used in 3 multicenter studies [24], [25], [51] in order to eliminate the risk of contamination when filling the syringe manually.

### Disinfection of catheter hub and other injection-/connecting points

The preventive bundles of 19 studies [24], [25], [26], [27], [29], [35], [36], [40], [42], [44], [46], [49], [50], [51], [52] stressed the importance of disinfecting catheter-hub three-way valves and needle-free connection valves upon each direct manipulation (“scrub the hub”). However, the studies varied regarding antiseptics used and exact procedure. Many studies used CHG (mostly 2%) with or without isopropanol (70%) to disinfect hubs or injection points, and exposure time varied between 15 and 30 seconds. Seven studies did not state the exact drying time after disinfection [24], [27], [36], [40], [47], [50], [51]. Sannoh et al. [46] requested that the disinfectant should dry at least 30 seconds, Wirtschafter et al. demanded only 15 seconds [25].

### Provision of necessary medical devices and products on a trolley

In 13 studies [25], [29], [30], [38], [42], [43], [46], [47], [48], [49], [50], [51], [52], a CVC trolley was present which provided all necessary medical products for catheter placement or dressing change. According to Sannoh et al. [46], all multi-bed rooms are equipped with such a trolley.

### Specialized teams 

A team of staff members with special skills/training was established in 10 studies [25], [27], [28], [29], [35], [36], [38], [43], [47], [48], in order to implement the preventive measures correctly. This team was responsible for PICC placement in 7 studies [25], [27], [28], [29], [36], [38], [47]. In some studies [25], [28], [35], [38], [43], [47], [48], this team was also responsible for maintaining care, e.g., change of system or dressing, or was explicitly responsible for supervision/monitoring and documenting PICC maintenance care [27], [28], [38], [47].

### Endpoint CLABSI infection rates 

**Figure 1:**
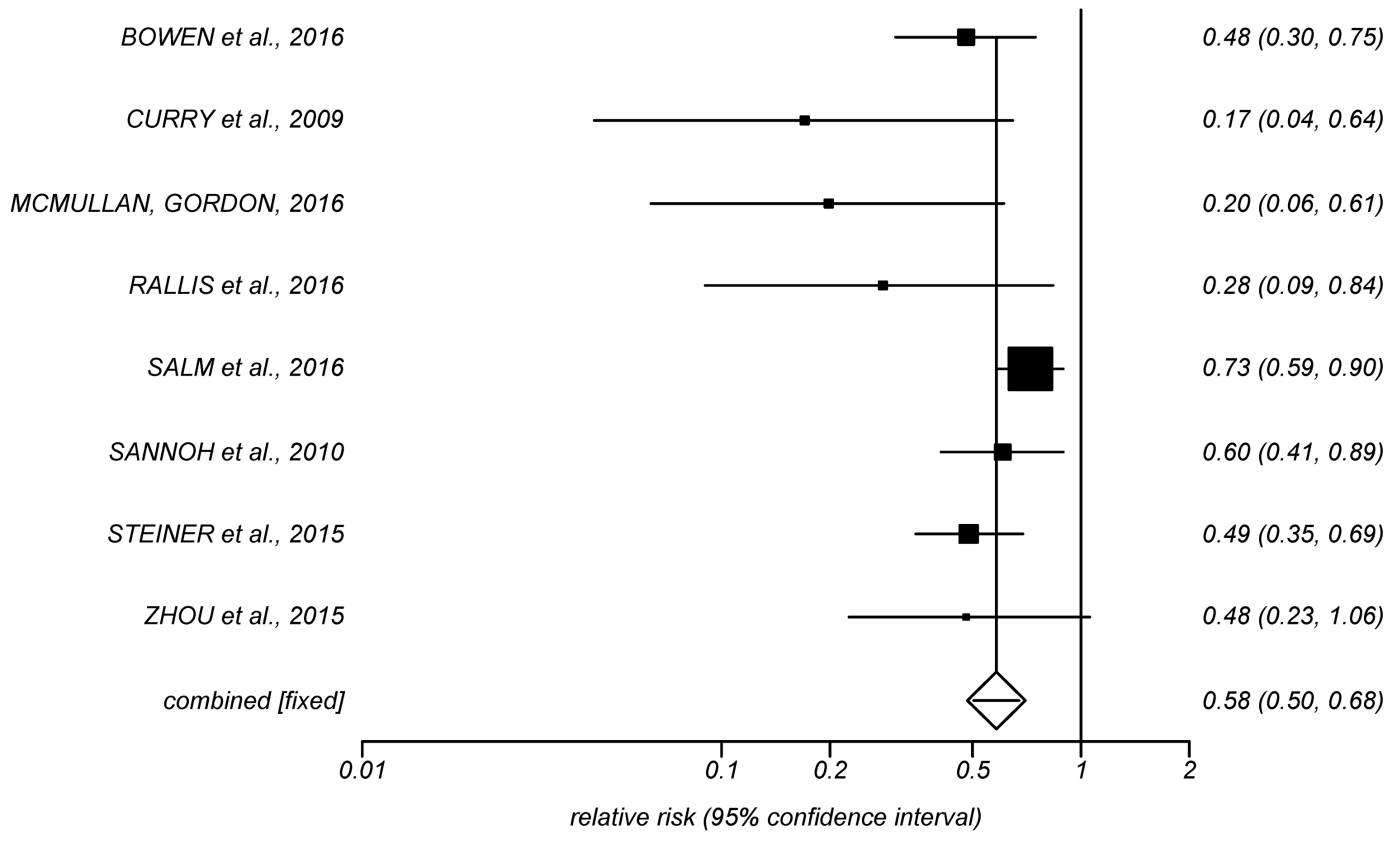
Forrest Plot, relative CABSI risk (incidences, 8 studies)

**Figure 2:**
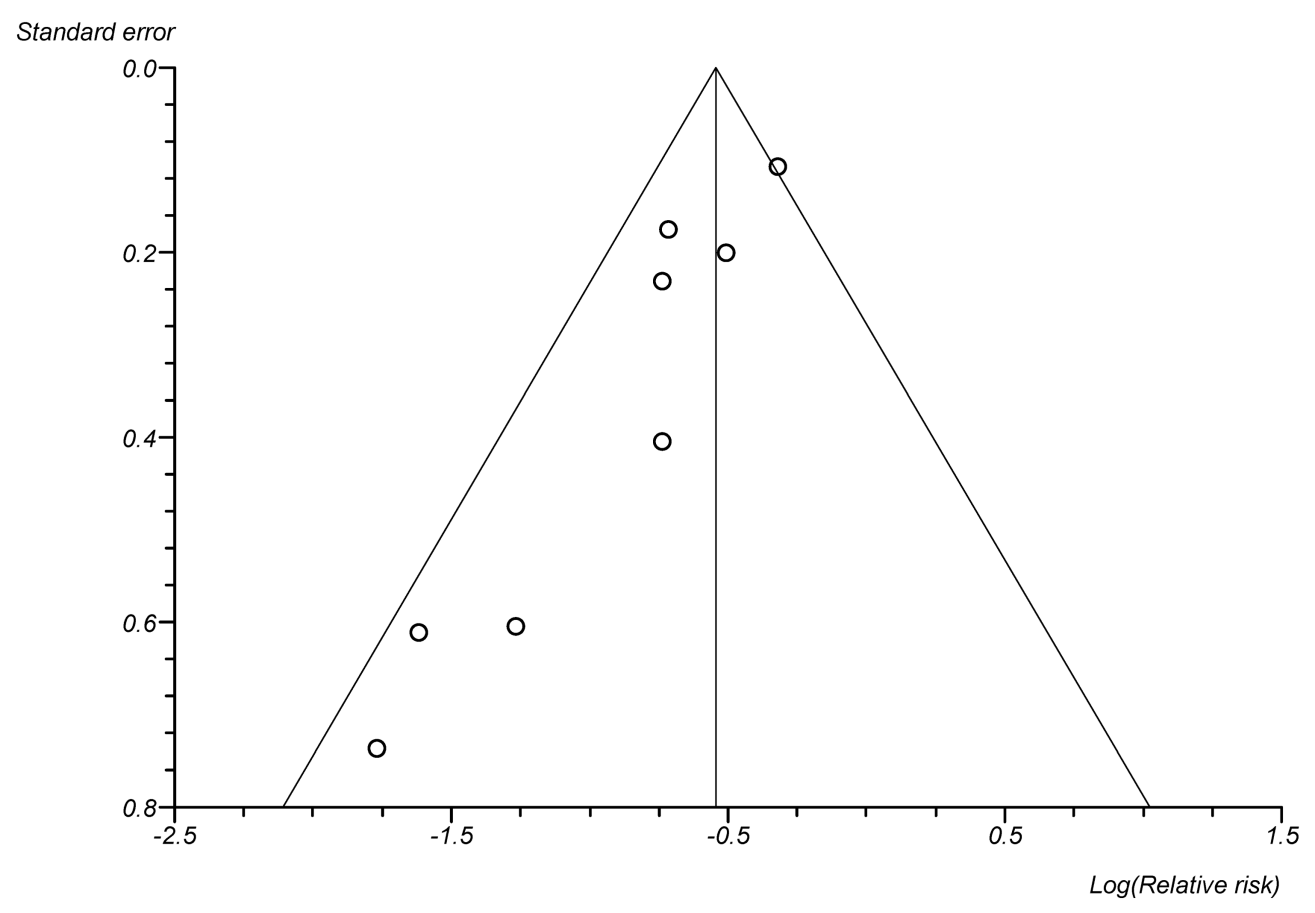
Funnel Plot, publication bias (CABSI incidences, 8 studies)

**Figure 3:**
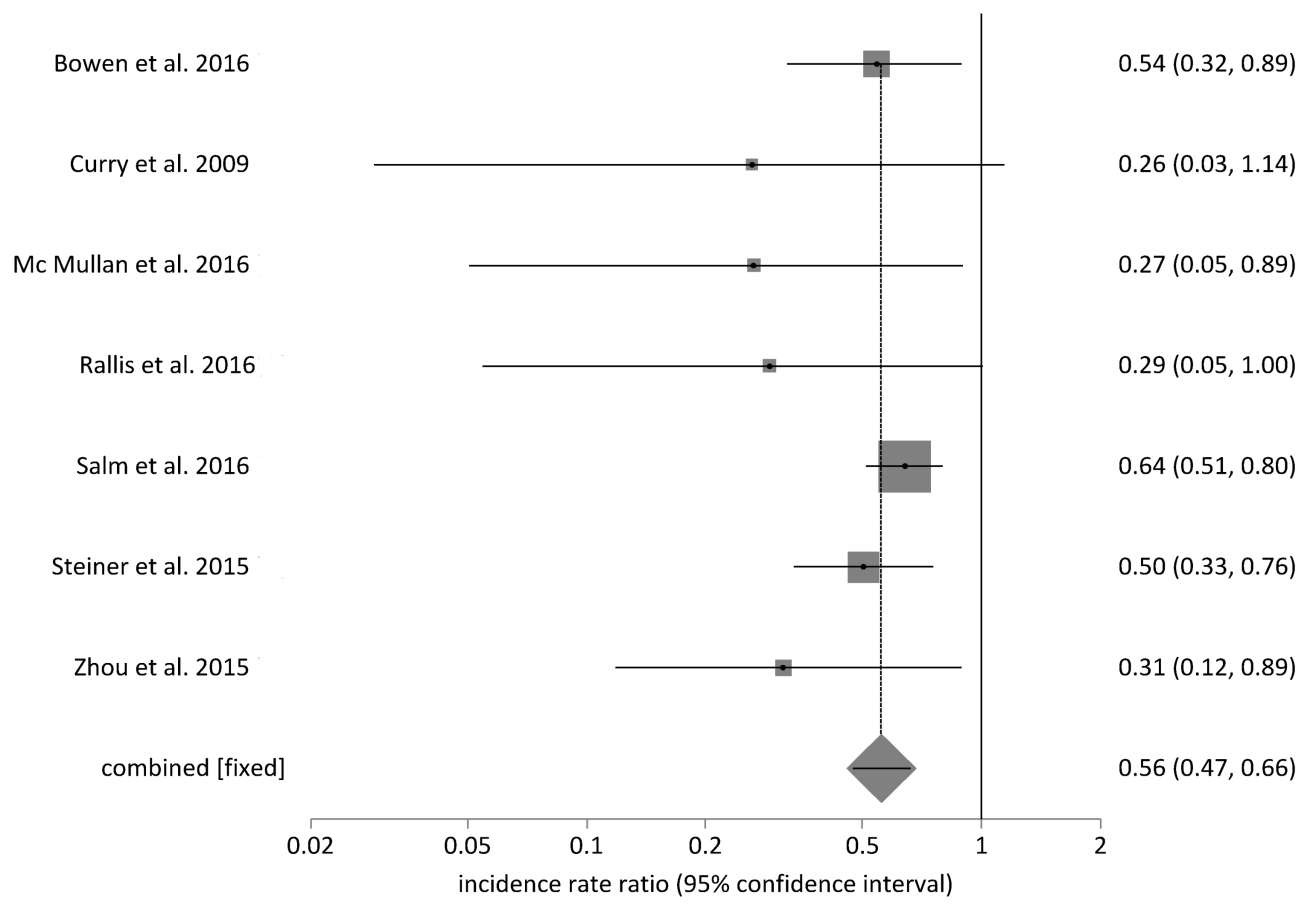
Forrest Plot, relative CABSI risk (incidence rate; 7 studies)

**Figure 4:**
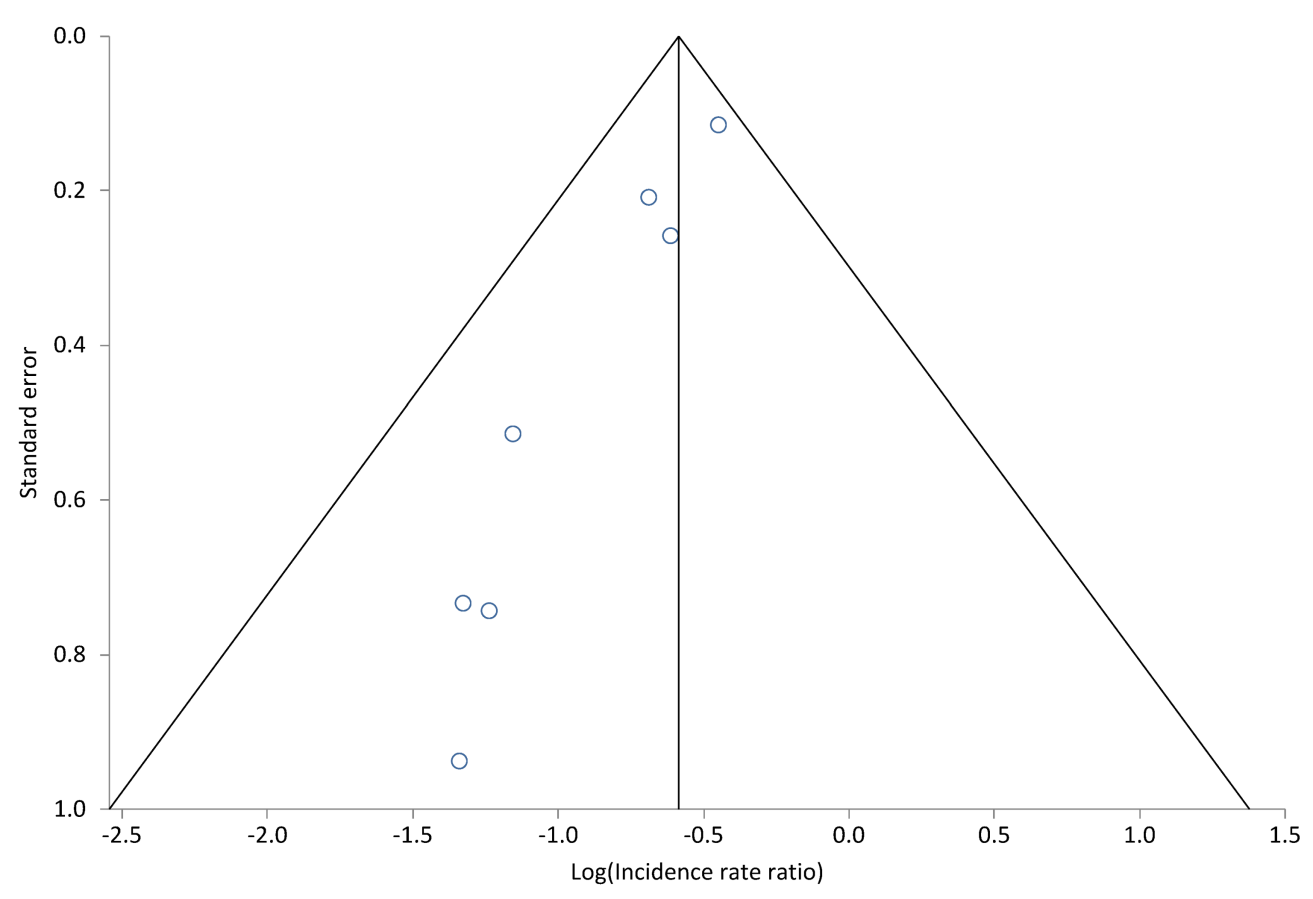
Funnel Plot, publication bias (CABSI incidences; 7 studies)

Table 2 (Tab. 2) shows the effects of preventive bundles on CLABSI rates. A significant reduction of CLABSI rates was found in 17 [26], [27], [28], [29], [30], [34], [35], [37], [38], [40], [41], [42], [45], [46], [49], [52], [53] of the 27 studies – we included Kilbride et al. [53], although they only investigated blood stream infections by CoNS. The relative risk after intervention was stated to be 0.17 to 0.75. This equals a decreasing probability of CLABSI of 25% to 83%. Six studies give examples of significant effects on a high initial rate between 11.6 and 16.7 CLABSI/1,000 utilization days [33], [34], [35], [38], [41], [45]. After intervening, the high initial rate drops to 0 to 5.2 CLABSI/1,000 utilization days. The findings of Kime et al. [33] show no statistical significance, while having a high clinical relevance with an initial rate of 15.6 CLABSI/1,000 utilization days and no CLABSI after intervention. Four additional studies [24], [43], [47], [48] exist which showed a non-significant decrease of CLABSI rate. Fisher et al. [50] report a reduction of CLABSI rate by 71% during a 10-month period. This was 19% in Piazza et al. [36] and 25% in Wirtschafter et al. [25]. Neill et al. [44] report that the number of events dropped by 92% in 5 years, from 6.08 to 0.45 per 1000 patient days. In addition, Figure 1 (Fig. 1) shows the results of a data meta-analysis from 8 studies which stated CLABSI incidences before and after intervention. Comparing groups before and after implementation of preventive bundles, the pooled relative risk (fixed effects, Mantel-Haenszel, Rothman-Boice) was at 0.58 (95% CI = 0.50–0.67) with moderate heterogeneity (I^2^ 48.8%; 95% CI 0–74.5%). Funnel plots (Figure 2 (Fig. 2)) of these studies and corresponding Egger’s tests for symmetry (–2.16; 95% CI –3.17 to –1.15; P=0.002) are indicating a possible publication bias in favor of a low pooled relative risk. Figure 3 (Fig. 3) shows the meta-analysis of 7 studies with a pooled rate ratio of 0.55 (95% CI 0.47–0.66; P<0.0001). The corresponding funnel plot also points towards a significant publication bias in favor of a low-pooled relative risk, as shown in Figure 4 (Fig. 4) (Egger Test –1.36; 95% CI –1.82 to –0.89; P=0.0006).

## Discussion

By analyzing and meta-analyzing 27 studies, this survey proves the benefit of preventive bundles on the prevention of CLABSI in premature NICU patients. This should motivate NICU teams to define local preventive bundles according to the latest KRINKO recommendations and to implement these measures sustainably [55]. In this context, the NEO KISS module provides a well-established and standardized instrument for the prospective surveillance of CLABSI in premature infants, which allows NICU teams to present and provide feedback on the long-term effects of preventive bundles to the entire team.

The problem of safe and effective skin antisepsis in very immature preterm infants of BW<1,500 g is still unresolved [56], especially in the first two weeks of life when the skin is extremely vulnerable. Many studies use different concentrations of chlorhexidine gluconate (CHG) for skin antisepsis in premature infants, despite the fact that CHG may cause serious local skin irritations [57] and is resorbed systemically [56], [58], [59], [60]. To date, it remains unclear which long-term consequences are caused by CHG exposure in premature infants. Based on an Orphan Drug approval of the European Medicines Agency (EMA), the KRINKO recommendations still name Octenidin 0.1% as the first-choice skin antiseptic. However, there is no commercially available ready-to-use product without 2% phenoxyethanol or 70% isopropanol. Even with Octenidin 0.1%, there is evidence of skin lesions in very immature preterm infants during the first 2 weeks of life [61]. Hence, the KRINKO currently recommends limiting exposed skin areas by using sterile drapings before skin antisepsis. 

Preventive bundles to reduce CLABSI in NICUs are part of an NI prevention master plan for premature infants [13]; see also Bowen’s initiative for quality improvement [49]. 

Besides a preventive bundle for PICCs and PVCs, the author’s list of recommended preventive measures comprises additional information on structural-organisational aspects (e.g., patient-related medical products/stethoscopes, processing medical traps, administering mother’s milk and probiotics, kangarooing, visitor regulations and antibiotic stewardship in NICUs). 

In Germany, there are additional measures in place such as weekly colonization screening to detect and stop the nosocomial transfer of antibiotic-resistant pathogens at an early stage [62], precautions for the aseptic reconstitution or preparation of medical products for parenteral use [63], [64], and concepts to decolonize premature infants colonized or infected with methicillin-resistant *S. aureus* [65].

## Limitations

The investigated studies differed regarding the implementation of preventive bundles and definition of endpoints (see Table 2 (Tab. 2)). The effect of different definitions can be made clear when we look at the consequences of the new 2008 definition of CLABSI caused by potential contaminants of blood culture, i.e., skin flora bacteria. According to the new CDC definition [54], coagulase-negative staphylococci (CoNS) must be detected in any case by means of two or more independently drawn blood cultures. Blood culture results reveal CoNS as the most frequent source of infection in premature NICU patients with late-onset sepsis diagnosed after the third day of life. Schulman et al. [52] describe a decrease of CoNS-caused CLABSI from 59% to 41% based only on the retrospective adaptation of CDC definitions after 2008. Accordingly, CoNS were not detected in two separately drawn blood cultures in 17% of CLABSI before 2008. Premature infants have a very low blood volume (100 ml/kg equalling 50 ml in an infant with a body weight of 500 g). Aerobic blood culture bottles are approved for this small blood volume, but fillings frequently fall short of the recommended minimum volume of 1 ml [66], [67], let alone drawing two such blood cultures of 1 ml each before starting an empirical antibiotic therapy. For the same reasons, it is not possible in neonatal intensive care to do routine parallel central and peripheral venous blood cultures in order to define the differential time to positivity. 

Relevant NEO KISS definitions, i.e., before the update in 2016, state *“A single proof of coagulase-negative staphylococci does not necessarily rule out the diagnosis of clinical sepsis. Clinical sepsis may be diagnosed even with one proof of CoNS in blood culture when classified as contamination while not meeting CoNS sepsis criteria but meeting criteria for clinical sepsis”*. A *“microbiologically confirmed sepsis with CoNS as only pathogen”* must be confirmed through at least one additional laboratory parameter and a minimum of two additional clinical criteria. To this extent the single proof of CoNS in the blood culture of a preterm patient with clinical sepsis may be assessed as contamination (*“clinical sepsis”*) or detection of a pathogen (*“microbiologically verified sepsis with CNS as only pathogen”*). Moreover, CLABSIs are not established as certain catheter-originating infections [66]. Sepsis was considered to be a CLABSI when the patient had a central vascular access within 48 hrs before infection or at the beginning of the infection, and when there was no other primary focus of infection defined by imaging or clinical evaluation. 

Semi-quantitative roll-plate culture of catheter tips [68] is not part of surveillance definitions for preterm infants, although significant growth (e.g., ≥15 CFU according to Maki’s method) points to the catheter as the probable source of the infection. CLABSI surveillance criteria are not decisive factors for clinical assessment of suspected late-onset infections. 

The benefit of a preventive bundle can only be assessed realistically when we know how many times the components are definitely accomplished. Most study protocols of this survey (20 of 27; 74%) included a verification of compliance, but methods of near-patient monitoring and feedback differed widely. Supervision of compliance with hand hygiene seems highly useful [69]. This also applies to other crucial checkpoints, such as skin antisepsis and maximum barrier measures for PICC placement, disinfection of hubs, needle-free connective valves and other injection points before each manipulation, or procedural details for dressing and IV system changes. Many multicenter studies obliged participating centers to supervise and secure compliance with preventive measures. 

Well-trained hygiene specialist staff are highly suitable for checking the compliance through aimed auditing of NICUs, but such staff with sufficient working time are not available everywhere. Checklists and especially a strict provision requiring two licensed nurses for all critical manipulations like dressing changes or line changes are useful. In some studies, there were specialist teams to perform placement and maintenance of central lines [25], [27], [28], [29], [35], [36], [38], [43], [47], [48], whereas most German NICUs aim at personal responsibility in letting nurses and doctors perform necessary actions autonomously. Considerable efforts are necessary for the new introduction of preventive bundles concerning knowledge transfer courses and training skills, which must be taken into account, e.g., in terms of working time when planning the practical implementation of such measures [55]. The scientific examination of preventive bundles is not suited to showing the specific benefit of individual bundle components. Nevertheless, merging individual measures of proven benefit in term of reducing infection risks may result in a higher overall effect on CLABSI rates.

In conclusion, our evaluation impressively confirms the benefit of preventive bundles regarding the prevention of CVC-associated infections in premature NICU patients. The heads of German NICUs should examine local preventive strategies according to KRINKO recommendations. Preventive bundles should be defined together with all involved professional groups and sustainably implemented in daily clinical routine.

## Abbreviations

- BW – birth weight
- CDC – Centers for Disease Control and Prevention, Atlanta, USA
- CLABSI – central line associated bloodstream infections
- CFU – colony forming units
- CHG – chlorhexidine gluconate
- CNS – coagulase-negative staphylococci
- CRBSI – catheter related blood stream infection
- CVC – central venous catheter
- ELBW – extremely low birth weight (<1,000 g)
- GA – gestational age
- ICU – intensive care unit
- KRINKO – German Commission for Hospital Hygiene and Infection Prevention affiliated to the Robert Koch Institute, Berlin, Germany
- LOS – late-onset sepsis
- NI – nosocomial infection
- NICU – neonatal intensive care unit
- PICC – peripherally inserted central venous catheter
- PVC – peripheral venous indwelling cannula
- UAC – umbilical artery catheter
- UVC – umbilical vein catheter
- VLBW – very low birth weight (<1,500 g)

## Notes

### Competing interests

The authors declare that there are no conflicts of interest. Prof. Simon is coordinator of the working group on neonatal intensive care of the German Commission for Hospital Hygiene and Infection Prevention affiliated to the Robert Koch Institute in Berlin, Germany. Prof. Geffers is leader of the NEO-KISS surveillance module of the German National Reference Center for Surveillance of Nosocomial Infections, Institute for Hygiene and Environmental Medicine at the Charité in Berlin, Germany.

### Acknowledgements

Our thanks go to all members of the working group on neonatal intensive care of the Commission for Hospital Hygiene and Infection Prevention, Dr. Jürgen Christoph, Prof. Dr. Christof Dame, Prof. Dr. Christian Gille, Prof. Dr. Christoph Härtel, Prof. Dr. Irene Krämer, Dr. Matthias Marschal, Prof. Dr. Andreas Müller, Prof. Dr. Mardjan Arvand and Vanda Marujo.

## Figures and Tables

**Table 1:**
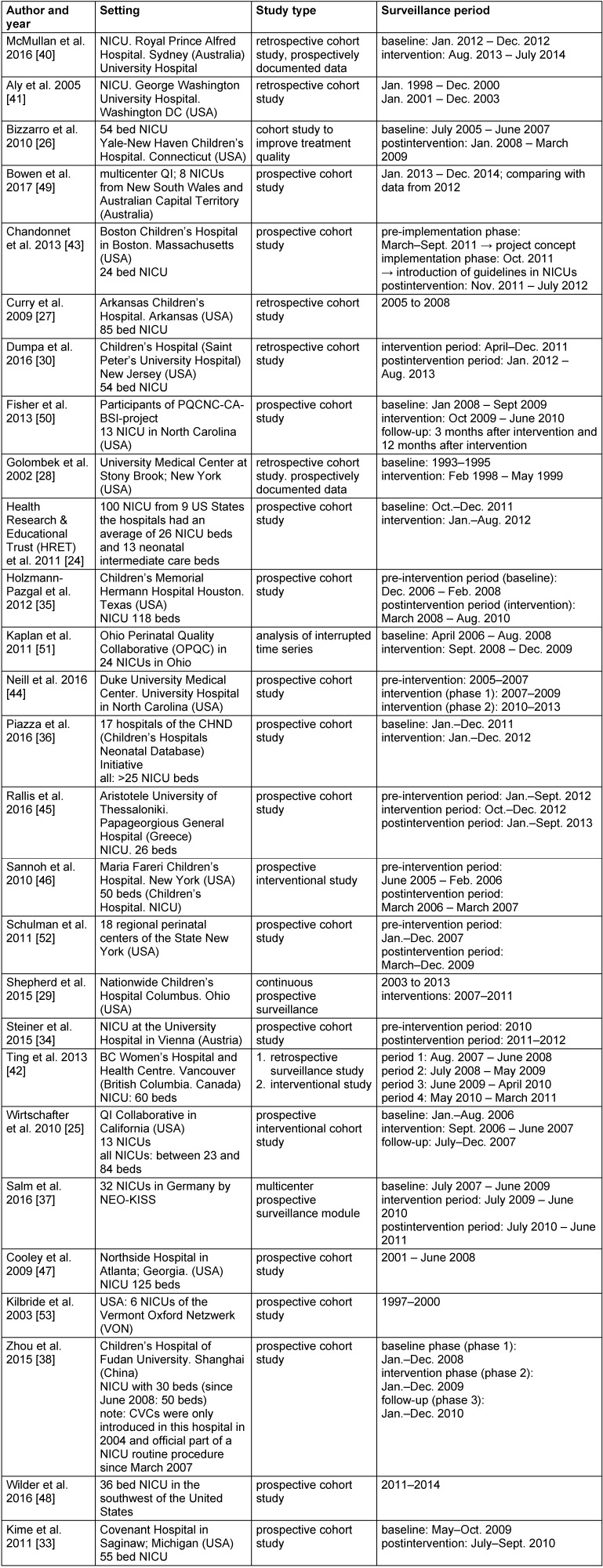
Included studies, setting, study type and surveillance period

**Table 2:**
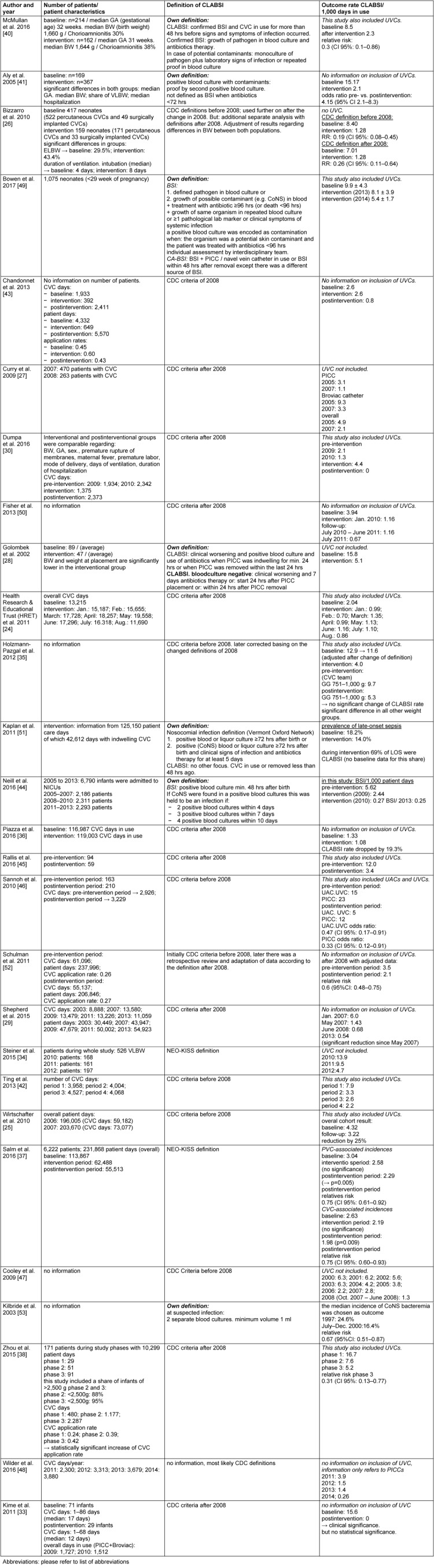
Included studies, number of patients, patient characteristics, outcomes
